# Mechanisms of local immunosuppression in cutaneous melanoma

**DOI:** 10.1038/sj.bjc.6603763

**Published:** 2007-06-12

**Authors:** M E Polak, N J Borthwick, F G Gabriel, P Johnson, B Higgins, J Hurren, D McCormick, M J Jager, I A Cree

**Affiliations:** 1 Translational Oncology Research Centre, Department of Surgery and Histopathology, Queen Alexandra Hospital, Southwick Hill, Portsmouth PO6 3LY, UK; 2 Department of Ophthalmology, Leiden University Medical Center, Leiden 2333 ZA, The Netherlands; 3 Department of Pathology, Institute of Ophthalmology, Bath Street, London EC1V 9EL, UK; 4 PIMHS University of Portsmouth, 2 King Richard Ist Road, Portsmouth PO1 2FR, UK

**Keywords:** melanoma, tolerogenic dendritic cell, FOXp3, IDO, TGF*β*2 immunosuppression

## Abstract

Cutaneous melanoma is highly immunogenic, yet primary melanomas and metastases develop successfully in otherwise immunocompetent patients. To investigate the local immunosuppressive microenvironment, we examined the presence of suppressor T lymphocytes and tolerising dendritic cells (DCs), the expression of immunosuppressive cytokines (IL-10, TGF*β*1 and TGF*β*2) and the enzyme indoleamine 2,3-dioxygenase (IDO) using qRT–PCR and immunohistochemistry in primary skin melanomas, negative and positive sentinel lymph nodes (SLN), and lymph nodes with advanced metastases. Our results indicate that tolerogenic DCs and suppressor T lymphocytes are present in melanoma at all stages of disease progression. They express transforming growth factor *β* receptor 1 (TGF*β*R1), and are therefore susceptible to TGF*β*1 and TGF*β*2 specifically expressed by primary melanoma. We found that expression of IDO and interleukin 10 (IL-10) increased with melanoma progression, with the highest concentration in positive SLN. We suggest that negative SLN contain immunosuppressive cells and cytokines, due to preconditioning by tolerogenic DCs migrating from the primary melanoma site to the SLN. In primary melanoma, TGF*β*2 is likely to render peripheral DCs tolerogenic, while in lymph nodes IDO and TGF*β*1 may have a major effect. This mechanism of tumour-associated immunosuppression may inhibit the immune response to the tumour and may explain the discrepancy between the induction of systemic immunity by anti-melanoma vaccines and their poor performance in the clinic.

Regulation of immune responses is essential to avoid autoimmunity, but there is a fine balance between switching off deleterious immune responses and suppressing beneficial ones. This is particularly true in cancer, where suppression of the immune response assists tumour growth and metastases. Both CD8+ and CD4+ T lymphocyte subsets can downregulate immune responses via direct interactions with antigen-presenting cells (APC), or through APC-independent pathways, for example by secreting immunoregulatory cytokines (Filaci and Suciu-Foca, 2002; Jonuleit and Schmitt, 2003). These regulatory or suppressor lymphocytes can be identified by expression of the transcription factor FoxP3 (Filaci and Suciu-Foca, 2002; Suciu-Foca *et al*, 2005).

Dendritic cells are involved in immunosuppression in numerous ways, both at the level of clonal deletion in the thymus and in the development of peripheral tolerance. Dendritic cell-derived immunosuppression is antigen dependent and can result either from antigen presentation by immature dendritic cells (DCs) that lack sufficient accessory molecules, lack of proinflammatory cytokines (Lutz and Schuler, 2002; Enk, 2005), the involvement of immunoglobulin-like transcripts 3 and 4 (ILT3 and ILT4) on tolerogenic DCs and secretion of immunosuppressive agents, such as indoleamine 2,3-dioxygenase (IDO), interleukin 10 (IL-10) and transforming growth factor *β* (TGF*β*) (Manavalan *et al*, 2003; Enk, 2005). Additionally, immature DCs can differentiate into tolerogenic DCs under the influence of IL-10, TNF*α* and regulatory T lymphocytes (Enk, 2005). Interactions between immunosuppressive T lymphocytes and tolerogenic DCs are numerous, and generally result in cytokine-driven immunomodulatory responses (Manavalan *et al*, 2003; Enk, 2005; Suciu-Foca *et al*, 2005).

Cutaneous melanoma has long been recognised as an immunogenic tumour that successfully evades immune recognition while paradoxically, spreads through the lymphatic system. As yet no truly successful anti-melanoma vaccine has been developed, although various strategies have been tested. In general, numerous tumour-reactive T lymphocytes can be detected in the patient's blood following vaccination, but tumour growth is unaffected (Arienti *et al*, 1996; Lee *et al*, 1998; Baurain *et al*, 2000; Appay *et al*, 2006). One explanation is that the tumour microenvironment is so immunosuppressive that any incoming effector cell is impeded (Appay *et al*, 2006). Proposed mechanisms for melanoma-derived immunosuppression include the secretion of TGF*β*, IL-10 and IDO, which have been found in patients' blood, primary melanomas and sentinel lymph nodes (SLN) (Reed *et al*, 1994; Real *et al*, 2001; Lee *et al*, 2003, 2005; Redondo *et al*, 2003). Additionally, levels were found to correlate with tumour progression and invasiveness (Reed *et al*, 1994; Conrad *et al*, 1999). This suppression is likely to be local to the tumour, but could affect immune cells in draining lymph nodes as well.

The role of TGF*β* in tumour growth promotion and immunosuppression has been extensively studied. This cytokine can be produced by tumour cells, and can act in an autocrine and paracrine manner (Clark and Coker, 1998). Immune cells such as T lymphocytes and DCs have also been reported to secrete various isoforms of TGF*β*, and the presence of this cytokine promotes an immunosuppressive phenotype in both of these cell types (Chen and Wahl, 2003; Ghiringhelli *et al*, 2005). Interleukin 10 exerts suppressive effects on a wide range of immune cells, including T lymphocytes and DCs. It can be produced by regulatory T lymphocytes and by tumour cells (Enk, 2005). Indoleamine 2,3-dioxygenase is an enzyme produced mainly by APC of myeloid origin that suppresses T lymphocyte-related antigen-specific immune responses (Mellor and Munn, 2004). It has also been associated with disease progression in various tumour types, including colorectal cancer (Brandacher *et al*, 2006), haemangioma (Ritter *et al*, 2003) and melanoma (Harlin *et al*, 2006).

As both T lymphocytes and DCs are present in close proximity to melanoma and even within the tumour mass itself (Vermi *et al*, 2003; Bernsen *et al*, 2004; Polak *et al*, 2005), they are directly exposed to tumour-derived immunosuppressive cytokines. Indeed, alterations in DC numbers and maturity have been reported in SLN compared with non-SLN (Lana *et al*, 2001; Botella-Estrada *et al*, 2005). To investigate this further, we have compared matched samples of primary skin melanoma and negative and positive SLN for evidence of immunosuppression. We have used quantitative PCR and immunohistochemistry (IHC) to enumerate the cytokines and immunosuppressive molecules within tumour and draining lymphoid tissue to determine the extent of immunosuppression in these tissues and to identify the key components.

## MATERIALS AND METHODS

### Patients and samples

Fifteen primary melanoma samples, 22 matching SLN and 19 lymph nodes with melanoma deposits from a separate cohort of patients were included in the study. In analysed positive SLN, the melanoma micrometastases were present, while in lymph nodes with melanoma deposit, the tumour replaced more than 75% of the lymph node. Among these, we identified 11 matched samples of individual progressing melanomas, including three primary skin melanomas for which both negative and positive SLN were available. The remaining eight matched pairs comprised seven pairs of skin melanoma and negative SLN and one additional pair of negative and positive SLN (data not shown). As a cancer unrelated control tissue, we used eight pairs of tonsils routinely removed in therapeutic tonsillectomies. All samples analysed by qRT–PCR and IHC came from formalin-fixed paraffin-embedded tissue (FFPE), obtained from the Portsmouth Hospitals archive. The patients gave their consent for the use of archival tissue for research purposes and the study was conducted according to MultiCentre and Local Research Ethics Committee regulations. The choice of a particular block was made on the basis of careful microscopic evaluation.

### RNA extraction and preparation of cDNA

Five 10 *μ*m sections were cut from each block for RNA recovery. The tissue was dewaxed and rehydrated by sequential washes first through xylene and then ethanol at 100, 90 and 70%. Ribonucleic acid was extracted using the Ambion RecoverAll Total Nucleic Acid Isolation kit (no. 1970, Ambion, Huntington, UK) according to the manufacturer's protocol. To improve the yield of RNA, each sample was additionally purified in a filtration step before the extraction, using Nucleospin filter columns (Nalgene, VWR international, Leicestershire, UK), and microcentrifuged at 13 000 g for 1 min. The flow-throughs were collected and RNA extracted according to the Ambion protocol. The quantity and purity of extracted RNA was assessed using a NanoDrop-2000 spectrophotometer. Based on these measurements, the RNA sample concentration was adjusted to 100 ng *μ*l^−1^ and aliquoted into RT reaction tubes, with a separate tube for the RT-negative control mix. The RT master mix was prepared at twice the concentration using the ABI High-Capacity cDNA Archive kit (Applied Biosystems, Warrington, UK) and added to prepared RNA aliquots. The RT reaction was carried out in a pre-programmed Hybaid OMN-E Thermal Cycler, in a two-step reaction, 10 min at room temperature followed by 120 min at 37°C.

### The FFPE tissue PCR reaction

The PCR reaction was carried out using the Taqman primer probe system, suitable for FFPE tissue, as primers are designed to flank regions of nucleic acid strands smaller than 150 bases in length. To ensure that our quantitative results were RNA damage independent, all of the data were analysed as a ratio between target gene expression and least variable housekeeping gene expression, assuming that any RNA damage due to fixation problems is random (Rupp and Locker, 1988; Godfrey *et al*, 2000; Cronin *et al*, 2004) and would affect the least variable housekeeping gene to the same degree as target genes. After testing four different housekeeping genes, HMBA was selected as the control housekeeping gene for tumour samples because its cycle threshold value showed the least variation between samples and its efficiency closely matched the transcription efficiency of the target genes. As the signal threshold cycle (CT) is often delayed in cDNA from FFPE material the standard Taqman PCR protocol was extended to 50 cycles. The best housekeeping gene CT was used as a sample inclusion criterion: all samples with CT above 37.5 were excluded from further analysis. We assessed the intra and inter-assay reproducibility in preliminary experiments, and as the results were satisfactory (difference in CT between triplicate wells lower than 0.5 CT), we reduced the repetitions to single-well measurements.

The PCR reaction was carried out using the Taqman primer probe system (Applied Biosystems), optimised for short mRNA sequences, and thus optimal for FFPE material. The Taqman primer probe FAM490-conjugated reagent was diluted 1 : 10 with Taqman Master Mix (both from Applied Biosystems) and 11 *μ*l of the mix was distributed into a 96-well PCR plate. The cDNA concentration was measured using a NanoDrop 2000 spectophotometer, adjusted to 300 ng *μ*l^−1^ and 9 *μ*l added to each well. We tested the following target genes: the cytokines TGF*β*1, TGF*β*2 and IL-10 (Taqman primer probes codes: Hs00171257, Hs00234244 and Hs00174086, respectively), the enzyme IDO (Hs00158032), two membrane-bound molecules, ILT3 and ILT4 and FOXp3, a nuclear transcription factor (Hs00429000, Hs00275975 and Hs00203958). The plate was designed to accommodate all the target genes, best housekeeping gene, sample RT-negative controls and no template controls. To ensure identical reaction conditions for matched-pair samples, all samples from one patient were run in the same PCR plate. For each run, a matching well factor plate (BioRad well factor reagent; BioRad, Herts, UK) was diluted 1 : 10 in nuclease-free water (Promega, Southampton, UK). Both loaded plates were spun down to remove any air bubble. A BioRad quantitative PCR thermal cycler (I-cycler) was used together with BioRad software. The standard Taqman protocol comprising well factor plate measurements (six cycles), followed by plate replacement and enzyme activation, 2 min at 50°C, and 10 min double-stranded cDNA denaturation at 95°C, was extended from 40 to 50 cycles of repeated 15 s per 95°C and 1 min per 60°C incubations.

### qPCR data analysis

Before carrying out data analysis on qRT–PCR results, amplification plots were checked to confirm that housekeeping, target genes and negative controls appeared as expected. The best housekeeping gene CT was determined and all samples for which the best housekeeping gene CT exceeded 37.5 cycles were excluded from further evaluation. For all the genes for which the CT exceeded 40 cycles, the PCR efficiency analysis was repeated, and only samples whose amplification plot slopes did not differ from the best housekeeping gene CT were included in further analysis. Relative target gene expression was then calculated according to the expression 2^−ΔCT^, where ΔCT equals the difference between target gene CT and reference gene (best housekeeping gene) CT. The median relative gene expressions of each target were used in further comparisons. The gene expression differences between groups were tested in one-parameter multivariant analysis ANOVA. Separately, comparisons within two groups of matched samples were performed, namely three complete matched samples, including primary melanoma, negative and positive SLN and seven negative SLN *vs* positive SLN matched pairs. We used the Wilcoxon matched pair test for statistical analysis.

### Immunohistochemistry

To assess whether the gene expression data matched the actual protein production, we performed immunostaining for TGF*β*2, TGF*β*R, IDO and FOXp3 on skin and SLN sections. The primary antibodies were purchased from Abcam (Cambridge, UK) (TGF*β*2, FOXp3), Novocastra (Newcastle upon Tyne, UK) (TGF*β*R) and Chemicon (Chandlers Ford, UK) (IDO, rabbit polyclonal). Enzymatic digestion (TGF*β*2), microwave pretreatment (IDO) and pressure cooking were included for antigen retrieval purposes. To evaluate the presence of DCs in the tissues examined, we used the anti-FXIIIa antibody from Calbiochem (Nottingham, UK), (after pressure cooking pretreatment), as this is a standard dermal DC marker antibody in our laboratory (Polak *et al*, 2005). Double staining for FXIIIa and IDO performed in chosen SLN enabled us to identify cells that secreted IDO. To avoid confusion with melanin, we used the red chromogen, Fuchsin, for visualisation of positive staining. For double staining, performed in SLN without significant melanoma micrometastases, we used combined DAB and Fuchsin visualisation systems.

## RESULTS

### Stage-related expression of immunosuppressive cell markers and soluble molecules

#### Immunological cell infiltrates

**Figure 1:**
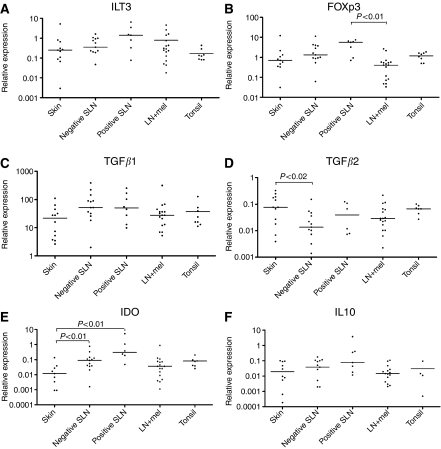
Stage-related expression of immunosuppressive molecules in cutaneous melanoma. The graphs in the panel show expression of (**A**) ILT3, a tolerogenic DC marker, (**B**) FOXp3, a regulatory and suppressive T lymphocyte marker, (**C, D**) two isoforms of TGF*β*, an immunoregulatory cytokine, (**E**) IDO, an immunoregulatory enzyme produced by APC of myeloid origin and (**F**) IL-10, an immunosuppressive cytokine produced by T lymphocytes and tolerogenic DCs at four stages of melanoma progression, that is, primary skin melanoma, negative SLN, positive SLN and lymph nodes with advanced melanoma metastases. The dots indicate relative expression of a target gene in comparison with the reference gene (HMBS) calculated as 2^−ΔCT^, where ΔCT=CT_target_−CT_HMBS_. Each dot represents the value for one sample, and the horizontal line indicates the median value for the site. The expression of ILT3, FOXp3, IL-10 and IDO increases with melanoma progression into SLN, with the peak concentration in the positive SLN, while concentration of TGF*β*1 remains stable between negative and positive SLN, after initial intense increase in negative SLN. The concentration of TGF*β*2 is the highest in primary skin melanoma, and is restored in SLN only after the invasion of melanoma. The *P*-values for statistically significant differences in molecule expression are shown where relevant. The last set of data presents expression of tolerogenic cell markers in tonsils, used as control lymphatic tissue.

**Figure 2:**
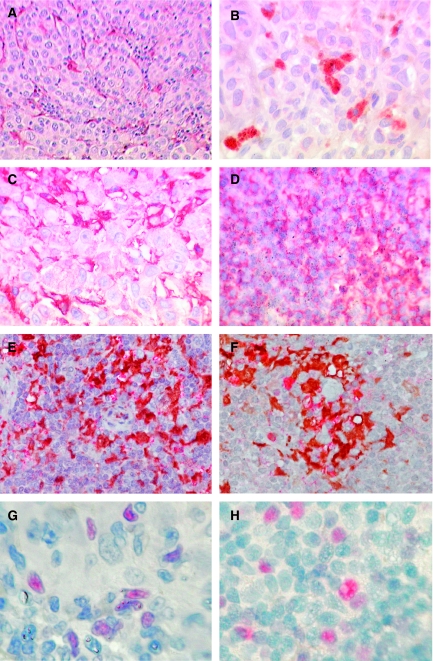
Expression of immunosuppressive cytokines and tolerogenic cell markers in melanoma at different stages of progression. To confirm the PCR-detected expression of mRNA for the cell markers and immunosuppressive cytokines examined, we performed IHC on sections from the same block as that used for RNA extraction. To avoid confusion with melanin, a red chromogen (Fuksin, Dako, UK) has been used and representative photographs taken to illustrate the findings. The images are as follows, with the original magnifications in parentheses: (**A**) FXIIIa+ cells within the tumour tissue in a lymph node largely replaced by metastatic melanoma (× 400). (**B**) TGF*β*2 expression in by melanoma cells in primary cutaneous melanoma (× 400). (**C**) TGF*β*R1 expression by lymphoid cells in primary skin melanoma (× 400). (**D**) TGF*β*R1 expression in by lymphoid cells in an SLN positive for a melanoma micrometastasis (not shown) (× 400). (**E**) Double staining for IDO (red) and FXIIIa (brown) in a negative SLN (× 400). (**F**) Double staining IDO (red) FXIIIa (brown) in a melanoma-positive SLN (× 400). (**G**) Expression of FoxP3+, a nuclear transcription factor regulatory and suppressive T lymphocyte marker, in primary skin melanoma (× 1000). (**H**) Expression of FoxP3+ lymphocytes in a melanoma-positive SLN (× 1000).

In initial experiments, cDNA derived from unrelated primary cutaneous melanoma, positive and negative SLN, lymph nodes with melanoma deposits and tonsil were compared. To evaluate the subtypes of immunosuppressive cells associated with progression of cutaneous melanoma, we measured FOXp3, a transcription factor found in both suppressor and regulatory T lymphocytes (Suciu-Foca *et al*, 2005), and ILT3 and ILT4 that are associated with tolerogenic DCs (Manavalan *et al*, 2003). High levels of FoxP3 and ILT3 expression were observed in more than 85% of the samples in each category, including the primary tumour, and were expressed at levels roughly equivalent to the housekeeping gene (Figure 1; HK=1.00). Expression of ILT4 was generally about a 100 times lower and often at borderline detection levels, and did not differ markedly between sites (data not shown). Both FoxP3 and ILT3 showed similar expression patterns and were detected in all four melanoma sites, but were highest in positive SLN (Figure 1A and B). The presence of FoxP3-positive lymphocytes was confirmed by IHC. FoxP3-positive cells were present as single positive cells within the lymphatic tissue in SLN as well as among tumour cells in primary melanoma of the skin (Figure 2C).

#### Expression of immunosuppressive cytokines and IDO

As secreted soluble molecules can promote tumour-derived immunosuppression, we examined the expression of two isoforms of TGF*β*, which can be produced by melanoma cells and by suppressor or regulatory lymphocytes; IL-10, produced by DCs and suppressive or regulatory T lymphocytes and IDO, produced mainly by APC of myeloid origin. Expression of all four molecules was detected in all sites examined: expression of the two isoforms of TGF*β* being the most ubiquitous (TGF*β*1 was present in 100% of cases, TGF*β*2 in 92.3% of the negative SLN and in 100% in the three other categories). Transforming growth factor *β*1 had by far the highest overall expression and was the most uniform at different stages of melanoma progression (Figure 1C). Despite a noticeable increase in TGF*β*1 expression after progression into the negative SLN, no further enhancement was observed with melanoma metastasis to the lymph nodes. Transforming growth factor *β*2 expression was highest in primary skin melanoma and lowest in negative SLN, indicating its release by tumour cells themselves. This was confirmed by IHC, which showed that TGF*β*2 is primarily tumour cell associated (Figure 2). Expression of IDO was detected in 75% of primary cutaneous melanoma samples and positive SLN, and in 100% of negative SLNs and lymph nodes with extensive melanoma metastasis. Interleukin 10 was detected in between 83 and 88% of cases at each stage. As with ILT3 and FOXp3, expression of both IL-10 and IDO increased with melanoma progression from skin into the lymph nodes, the peak occurring in positive SLN (Figure 1E and F). This was most striking for IDO, where levels increased by a factor of four between skin and negative SLN, and increased again by a similar amount between negative and positive SLN. Expression of IDO by FXIIIa-positive DCs was confirmed by IHC (Figure 2D).

In lymph nodes with advanced metastasis, there was generally a decrease in the levels of all molecules investigated, although these remained detectable and were in some cases highly expressed. This observation might reflect differences in the ratio of lymphoid *vs* tumour tissue in these samples although IHC showed both FoxP3 and tolerogenic DCs to be present in both tissue compartments.

#### Expression of immunosuppressive molecules in tonsil

As a control, we examined expression of all of the markers associated with immunosuppression in tonsils. These were chosen because of their accessibility and because they are associated with the mucosal immune system, and might therefore naturally be expected to have an immunosuppressed phenotype. We observed less variation between samples in tonsil compared with melanoma. All of the markers investigated were also found in tonsil, although ILT4 was again present at very low levels. Median expression of FOXp3, ILT4 and IDO was comparable in tonsils and in negative SLN, whereas expression of ILT3 was higher in negative SLN (*P*=0.025; Figure 1A). Median levels of both TGF*β*1 and IL-10 were higher in negative SLN than in tonsils (the difference statistically significant for IL-10, *P*=0.044; Figure 1F), while expression of TGF*β*2 was highest in tonsil (Figure 1D; *P*=0.012).

### Analysis of matched melanoma samples

**Figure 3:**
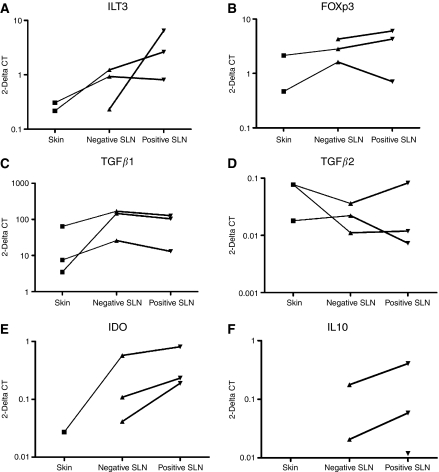
Changes in expression of immunosuppressive molecules during the disease progression in three matched melanoma samples. The graphs in the panel indicate changes in the expression of (**A**) ILT3, (**B**) FOXp3, (**C**) TGF*β*1 (**D**) TGF*β*2, (**E**) IDO and (**F**) IL-10 at three stages of melanoma progression, that is, primary cutaneous melanoma, negative SLN and positive SLN, each data set obtained from matched sites from one patient. Values on the *y*-axis represent relative expression of a target gene in comparison with the reference gene (HMBS) calculated as 2^−ΔCT^, where ΔCT=CT_target_−CT_HMBS_. The values of relative expression of DC and T lymphocyte markers (ILT3 and FOXp3) increase with melanoma progression in two cases, whereas expression of IDO and IL-10 increase in all three cases. The changes in expression of TGF*β*1 and TGF*β*2 are negatively correlated, and while the value for TGF*β*2 increases with melanoma invasion to SLN, expression of TGF*β*1 decreases in positive SLN.

**Figure 4:**
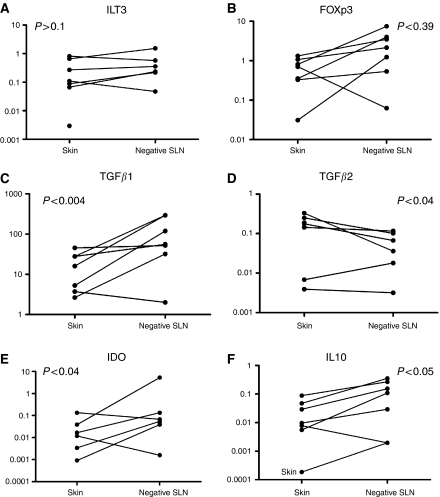
Changes in expression of immunosuppressive molecules in seven matched samples of primary skin melanoma and corresponding negative SLN. The graphs in the panel indicate changes in expression of (**A**) ILT3, (**B**) FOXp3, (**C**) TGF*β*1 (**D**) TGF*β*2, (**E**) IDO and (**F**) IL-10 in seven matched pairs of skin and negative SLN from melanoma patients. Values on the *y*-axis represent relative expression of a target gene in comparison with the reference gene (HMBS) calculated as 2^−ΔCT^, where ΔCT=CT_target_−CT_HMBS_. The *P*-values given above each set of data are obtained from a Wilcoxon paired test analysis, and are significant for all the molecules except ILT3. The observations of note are the very intense increase of TGF*β*1 expression in negative SLN and disproportional increase of IDO expression (median 29-fold) in comparison with stable ILT3 expression.

The general trend observed in consecutive stages of melanoma was a gradual build up of the immunosuppressive markers ILT3, FOXp3, IL-10 and IDO, with peak expression occurring in positive SLN. This was in contrast to TGF*β*1 that remained high throughout and TGF*β*2 that was highest in the primary tumour. To verify these observations, we identified and examined melanoma samples from the same patients, three of which included skin, negative and positive SLN (Figure 3) and seven additional pairs of matched skin and negative SLN samples (Figure 4).

The trends observed previously were even more pronounced in matched pair samples. We observed higher expression of ILT3 (*P*<0.112), FOXp3 (*P*<0.039), IDO (*P*<0.037) and IL-10 (*P*<0.047) in negative SLN compared with skin samples (eight matched pairs in total for ILT3 and nine matched pairs in total for FOXp3, IDO and IL-10; Figures 3 and 4). In the three sets of samples containing positive SLN, the expression of all four molecules further increased in positive SLN (Figure 3).

It is of note that markers indicative of immunosuppression are also seen in negative SLN. In particular, the expression of IDO increased between primary cutaneous melanoma and negative SLN by median 29.11-fold in five out of seven cases examined (*P*<0.037), and IL-10 by median 6.37-fold (*P*<0.047).

TGF*β*1 was also increased in the matched negative SLN compared with skin samples (Figures 3 and 4), with a median fold increase of 10.55 (*P*<0.004). This was followed by a noticeable drop in positive SLN in all three of the matched series of samples (Figure 3). In contrast, relatively high levels of TGF*β*2 in skin were followed by a drop in the negative SLN in all but one of the pairs examined, although the magnitude was far less pronounced (median value 2.46; *P*<0.04).

#### Activation status of suppressor lymphocytes and tolerogenic DCs

Direct comparisons of immune cell infiltrates in skin, SLN and lymph nodes with advanced metastasis are difficult due to differences in the relative amounts of lymphatic tissue they contain. However, by comparing changes in the levels of each of the markers of immunosuppression at each of the stages of the disease it is possible to gain a better insight into how immunosuppression might develop as the tumour progresses (Table 1).

Dendritic cells present in negative SLN appear to be more tolerogenic than those within the primary tumour because, although the median expression of ILT3 remains stable, the expression of both IDO and TGF*β*1 increases (IDO 4.02, TGF*β*1 2.37; Table 1). This was also seen in the matched tissue analysis where the ratios of median relative expression in negative SLN and skin for ILT3, IDO and TGF*β*1 are respectively 2.37, 29.11 and 10.55. During progression from negative to positive SLN, expression of all markers of immunosuppression (apart from TGF*β*1) is increased. This is particularly true for ILT3 and IDO, which are increased approximately four-fold (Table 1). Suppressor/regulatory T cells, as measured by FOXp3, increase with progression from skin to negative SLN (1.74-fold; Table 1) and negative to positive SLN (4.29-fold; Table 1). This is not however reflected by a proportional increase in IL-10. Transforming growth factor*β*1, which can be a sign of T lymphocyte activity, is comparable to FOXp3 in negative SLN *vs* skin (2.37 and 1.74, respectively; Table 1), but expression of TGF*β*1 remains stable, with progression to positive SLN, while expression of FOXp3 increases 4.29-fold (Table 1). In lymph nodes with advanced metastasis there is less evidence of immunosuppression compared with earlier stages; a five-fold decrease in ILT3 expression and an almost 10-fold decrease in IDO expression (Table 1). When comparing matched skin-negative SLN pairs, the IL-10 and FOXp3 ratios are equivalent (6.37 and 5.91, respectively). The increase in TGF*β*1 relative expression in paired negative sentinel LN is 10.55-fold.

Expression of all the targets was greatly reduced in metastatic lymph nodes. Levels of TGF*β*1 and ILT3 were in fact very similar to those seen in the primary skin tumours. The expression of FOXp3 was twofold lower (ratio 0.53; Table 1); however, DCs in lymph nodes with advanced melanoma metastasis have a higher expression of IDO than primary skin tumours (ratio 1.63; Table 1).

#### Expression of TGF*β*R1

As TGF*β* seems to be an important component of the immunosuppressive character of melanoma, we investigated the presence of one of the receptors (TGF*β*R1), which is essential for TGF*β* signal transduction (Kaminska *et al*, 2005), in order to determine which cells were able to respond to the cytokine. Sections from all four sites were stained for TGF*β*R1 by IHC and evaluated. We found that staining for TGF*β*R1 was very robust, and that many, but not all, melanoma cells were positive for this receptor. The strongest expression of TGF*β*R1 was however observed on the immunological cell infiltrate, both in lymph node and in skin sections, rather than the melanoma cells (Figure 2B).

## DISCUSSION

As cutaneous melanoma occurs in the presence of an intact immune system, it must necessarily develop mechanisms to evade the immune response. These include the release of immunosuppressive cytokines that can further modulate antigen-specific immune cells. We have investigated the relationship between active immunosuppression and melanoma progression. We detected the presence of suppressor/regulatory T lymphocytes and tolerogenic DCs at all stages of melanoma development. FOXp3-positive, IL-10 producing T lymphocytes were recently reported in tumour, blood and ascites from patients with advanced melanoma, and were associated with melanoma-related immunosuppression (Appay *et al*, 2006; Cesana *et al*, 2006; Harlin *et al*, 2006). As well as FOXp3 we have also demonstrated expression of ILT3, a marker of tolerogenic DCs and have correlated markers of local immunosuppression with melanoma progression. The level of immunosuppression increased from skin, through negative SLN, with the peak concentration of lymphatic cell markers and immunosuppressive molecules in positive SLN. Direct comparisons of immune cell infiltrates in skin, SLN and lymph nodes with advanced metastasis are difficult due to differences in the relative amounts of lymphatic tissue they contain. However, by comparing changes in the levels of each of the markers of immunosuppression at each of the stages of the disease it is possible to gain a better insight into how immunosuppression might progress as the tumour progresses. Dendritic cells present in negative SLN appear to be more tolerogenic than those within the primary tumour because, although the median expression of ILT3 remains stable, the expression of both IDO and TGF*β*1 increases by as much as a factor of 30 in matched samples. Numbers of suppressor/regulatory T lymphocytes, as measured by FOXp3, almost double with progression from skin primary to negative SLN, and there are similar increases in IL-10.

This indicates an increase in tolerogenic DCs and T lymphocytes activity in comparison with primary skin melanoma. Strikingly, the expression of TGF*β*1 was not increased further once melanoma progressed into the lymph nodes, suggesting that the negative SLN is already immunosuppressed prior to melanoma metastasis.

In contrast, the much lower expression of TGF*β*2 is more closely related to the presence of melanoma cells, and this cytokine is nearly absent in negative SLN. Our finding supports the hypothesis that the presence of TGF*β*2 is correlated with melanoma progression (Reed *et al*, 1994). This suggests that TGF*β*2 (produced either directly by melanoma or by associated fibroblasts) and not TGF*β*1 might in fact be the primary melanoma-derived immunosuppressive factor. As nearly all of the infiltrating immune cells were positive for TGF*β*1R, they are potentially susceptible to TGF*β*-mediated immunosuppression. Data on the influence of TGF*β*2 on APC are scarce, but this cytokine has been shown to inhibit the antigen presenting abilities of epidermal APC in mice (Hosoi *et al*, 1993), and its presence correlated with tumour progression, despite peripheral antitumour T-cell activity in glioblastoma patients (Liau *et al*, 2005).

We suggest that TGF*β*2 produced by primary melanoma may convert peripheral DCs into tolerogenic cells. These could then migrate to the SLN, before any tumour spread takes place and prime the site for tumour growth, by producing IDO and TGF*β*1, and making the SLN an immunoprivileged site suitable for melanoma metastasis. In studies on mouse and human macrophages activated with IFN*γ* to secret IDO, TGF*β* was shown to inhibit IDO secretion (Bogdan and Nathan, 1993; MacKenzie *et al*, 1999). However, it is conceivable that tolerogenic DCs may react differently to DCs primed to activate immune responses. If this hypothesis is correct, it would mean that tolerogenic DCs generated in response to tumour derived cytokines actively support this process. Once the melanoma actually metastasises, and the SLN becomes positive, TGF*β*2 produced by invasive melanoma cells may further increase the immunosuppression, resulting in the development or attraction of suppressor/regulatory T cells, which in turn release immunosuppressive cytokines. As the lymph node metastasis develops, the measurements of immunosuppression markers significantly decrease. This may directly reflect the substantial increase of tumour infiltrates in comparison to residual lymphoid tissue, rendering the tissue type similar to primary melanoma rather than lymphatic tissue. Replacement of active lymph nodes by melanoma may also mean the strong local immunosuppression is no longer necessary. Nevertheless, despite cessation of TGF*β*2 production, the tolerogenic DCs are still present in numbers comparable with primary skin melanomas. Their immunosuppressive activity might then further support immune evasion by melanoma.

If tumour-associated peripheral DCs are indeed responsible for tumour-derived local immunosuppression, and enable the melanoma spread to preconditioned lymph nodes, this has considerable implications for the development of immunotherapy. Reversal of the tolerising effect exerted by DCs could be successful in preventing the onset of melanoma metastases, and substantially support action of other anti-melanoma vaccines. Indeed, vaccines using DC-specific immune adjuvant (particularly GM-CSF) can alter the number and activation status of DCs residing in melanoma SLN (Lana *et al*, 2001; Lee *et al*, 2005) and can induce the activation of peripheral blood lymphocytes resulting in some positive clinical responses (Vuylsteke *et al*, 2006). Ultimately, to enable any effector cell to act, either by means of an anti-melanoma immune vaccine or by the natural immune response, DC-mediated local immunosuppression in melanoma must be reversed. This hypothesis could further be investigated in a series of functional experiments with tolerogenic DCs and suppressive/regulatory T lymphocytes, to evaluate the exact mechanism of melanoma-associated early immunosuppression, and possibly discover ways to overcome it.

## Figures and Tables

**Table 1:** Ratios of median relative expression between different stages of melanoma progression

	**ILT3**	**ILT4**	**FOXp3**	**TGF**β**1**	**TGF**β**2**	**IDO**	**IL-10**
Negative SLN/skin	1.37	0.54	1.74	2.37	0.18	4.02	1.01
Positive SLN/negative SLN	4.00	2.84	4.29	0.97	2.90	3.46	2.00
LN+mel/positive SLN	0.20	0.16	0.07	0.55	0.74	0.12	0.20
LN+mel/skin	1.11	0.25	0.53	1.27	0.38	1.63	0.41

Abbreviations: IDO, indoleamine 2,3-dioxygenase; IL-10, interleukin 10; ILT3, immunoglobulin-like transcript 3; ILT4, immunoglobulin-like transcript 4; LN, lymph node; mel, melanoma; SLN, sentinel lymph nodes; TGF*β*, transforming growth factor *β*.
